# 4-[3-(Bromo­meth­yl)benz­yloxy]-3-methoxy­benzaldehyde

**DOI:** 10.1107/S1600536810011347

**Published:** 2010-03-31

**Authors:** Jin-Jian Wei, Lei Jin, Cheng-He Zhou, Yi-Yi Zhang

**Affiliations:** a Laboratory of Bioorganic & Medicinal Chemistry, School of Chemistry and Chemical Engineering, Southwest University, Chongqing 400715, People’s Republic of China; b School of Pharmaceutical Sciences, Southwest University, Chongqing 400715, People’s Republic of China

## Abstract

In the title compound, C_16_H_15_BrO_3_, the dihedral angle between the mean planes of the two benzene rings is 76.64 (2)°. In the crystal structure, there are weak π–π stacking inter­actions, with a centroid–centroid distance of 3.724 (3) Å, as well as an inter­molecular C⋯Br distance [3.495 (2) Å] which is slightly less than the sum of the van der Waals radii for these atoms.

## Related literature

For the applications of related compounds, see: Chen *et al.* (2001 ▶); Demestre *et al.* (2009 ▶); Liao *et al.* (2003 ▶); Xia & Hu (2004 ▶). For a related structure, see: Jin *et al.* (2009 ▶).
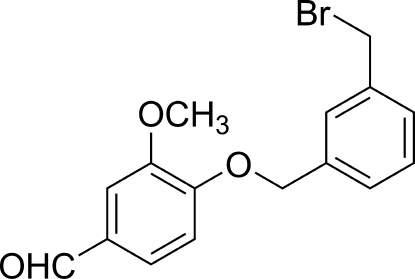


## Experimental

### 

#### Crystal data

C_16_H_15_BrO_3_
*M* *_r_* = 335.19
Monoclinic, 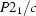

*a* = 14.275 (3) Å
*b* = 11.791 (2) Å
*c* = 8.7315 (17) Å
β = 95.671 (3)°
*V* = 1462.5 (5) Å^3^
*Z* = 4
Mo *K*α radiation
μ = 2.82 mm^−1^
*T* = 298 K
0.08 × 0.08 × 0.06 mm

#### Data collection

Bruker SMART CCD diffractometer
Absorption correction: multi-scan (*SADABS*; Sheldrick,1996 ▶) *T*_min_ = 0.798, *T*_max_ = 0.845
7566 measured reflections
2777 independent reflections
2014 reflections with *I* > 2σ(*I*)
*R*_int_ = 0.031

#### Refinement

*R*[*F* ^2^ > 2σ(*F* ^2^)] = 0.054
*wR*(*F* ^2^) = 0.150
*S* = 1.07
2777 reflections
182 parameters
H-atom parameters constrained
Δρ_max_ = 0.85 e Å^−3^
Δρ_min_ = −0.35 e Å^−3^

### 

Data collection: *SMART* (Bruker, 2001 ▶); cell refinement: *SAINT-Plus* (Bruker, 2001 ▶); data reduction: *SAINT-Plus*; program(s) used to solve structure: *SHELXS97* (Sheldrick, 2008 ▶); program(s) used to refine structure: *SHELXL97* (Sheldrick, 2008 ▶); molecular graphics: *SHELXTL* (Sheldrick, 2008 ▶); software used to prepare material for publication: *SHELXTL*.

## Supplementary Material

Crystal structure: contains datablocks I, global. DOI: 10.1107/S1600536810011347/lh5019sup1.cif

Structure factors: contains datablocks I. DOI: 10.1107/S1600536810011347/lh5019Isup2.hkl

Additional supplementary materials:  crystallographic information; 3D view; checkCIF report

## Figures and Tables

**Fig. 1.**
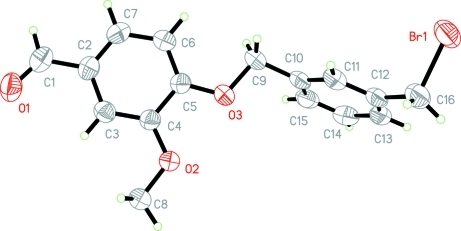
The molecular structure of (I), showing the atom-numbering scheme. Displacement ellipsoids are drawn at the 50% probability level.

## supplementary crystallographic information

### Comment

Vanillin (4-hydroxy-3-methoxybenzaldehyde) and its derivatives are important medicinal intermediates which are extensively employed to prepare bioactive compounds such as anti-hypertension compounds, diureses and deodorisers (Chen, *et al.*, 2001; Demestre, *et al.*, 2009; Liao, *et al.*, 2003; Xia, *et al.*, 2004). Recently, our research has been focused on the development of vanillin-derived azole drugs, and a nitroimidazole derivative has been reported (Jin, *et al.*, 2009). Herein we report the crystal structure of the title compound a potential intermediate for the synthesis of azole antifungal agents.

The molecular structure of the title compound is shown in Fig. 1. The dihedral angle between the mean planes of the two benzene rings is 76.64 (2)°. In the crystal structure, there are weak π–π stacking interactions where Cg···Cg(-x,2-y,1-z) = 3.724 (3)Å [Cg is the centroid of the C2-C7 ring] as well as an intermolecular C13···Br1(-x+1, 0.5+y, 2.5-z) distance [3.495 (2)Å] which is slightly less than the sum of the van der Waals radii for these atoms.

### Experimental

A suspension of 4-hyhydroxy-3-methoxybenzaldehyde (200 mg, 1.31 mmol) and anhydrous potassium carbonate (200 mg, 1.45 mmol, 1.2 equiv) in CH_3_CN (10 ml) was stirred for 30 min at 338 K, and then tetrabutyl ammonium iodide (TBAI, 5 mg) and 1,3-bis(bromomethyl)benzene (1 g, 3.78 mmol) were added. The resulting mixture was stirred for 5–7 h at 348–353 K (monitored by TLC, eluent, ethyl acetate/petroleum, V/V, 5/1). After the reaction solvent was evaporated under reduced pressure, water (10 mL) was added. The mixture was extracted with chloroform (3×10 ml). The organic layer was collected, dried over anhydrous Na_2_SO_4 _and evaporated under reduced pressure to give the crude product, which was purified by silica gel column chromatography (eluent, ethyl acetate/petroleum, V/V, 5/1) to afford the title compound (I). Single crystals were grown by slow evaporation of a solution of (I) in an ethyl acetate and petroleum mixture at room temperature.

### Refinement

Hydrogen atoms were placed in calculated positions with C—H = 0.93Å (aromatic), 0.97Å (methylene) and 0.96Å (methyl) with *Uiso*(H) = 1.2*Ueq*(C) (aromatic and methylene C) or 1.5*Ueq*(C) (methyl C).

### Crystal data

**Table:**

C_16_H_15_BrO_3_	*F*(000) = 680
*M**_r_* = 335.19	*D*_x_ = 1.522 Mg m^−^^3^
Monoclinic, *P*2_1_/*c*	Mo *K*α radiation, λ = 0.71073 Å
Hall symbol: -P 2ybc	Cell parameters from 2434 reflections
*a* = 14.275 (3) Å	θ = 2.3–24.0°
*b* = 11.791 (2) Å	µ = 2.82 mm^−^^1^
*c* = 8.7315 (17) Å	*T* = 298 K
β = 95.671 (3)°	Block, colourless
*V* = 1462.5 (5) Å^3^	0.08 × 0.08 × 0.06 mm
*Z* = 4	

### Data collection

**Table:**

Bruker SMART CCD diffractometer	2777 independent reflections
Radiation source: fine-focus sealed tube	2014 reflections with *I* > 2σ(*I*)
graphite	*R*_int_ = 0.031
φ and ω scans	θ_max_ = 25.7°, θ_min_ = 2.2°
Absorption correction: multi-scan (*SADABS*; Sheldrick,1996)	*h* = −17→17
*T*_min_ = 0.798, *T*_max_ = 0.845	*k* = −14→14
7566 measured reflections	*l* = −10→5

### Refinement

**Table:**

Refinement on *F*^2^	Primary atom site location: structure-invariant direct methods
Least-squares matrix: full	Secondary atom site location: difference Fourier map
*R*[*F*^2^ > 2σ(*F*^2^)] = 0.054	Hydrogen site location: inferred from neighbouring sites
*wR*(*F*^2^) = 0.150	H-atom parameters constrained
*S* = 1.07	*w* = 1/[σ^2^(*F*_o_^2^) + (0.0765*P*)^2^ + 1.2302*P*] where *P* = (*F*_o_^2^ + 2*F*_c_^2^)/3
2777 reflections	(Δ/σ)_max_ = 0.001
182 parameters	Δρ_max_ = 0.85 e Å^−^^3^
0 restraints	Δρ_min_ = −0.35 e Å^−^^3^

### Special details

**Table:**

Geometry. All esds (except the esd in the dihedral angle between two l.s. planes) are estimated using the full covariance matrix. The cell esds are taken into account individually in the estimation of esds in distances, angles and torsion angles; correlations between esds in cell parameters are only used when they are defined by crystal symmetry. An approximate (isotropic) treatment of cell esds is used for estimating esds involving l.s. planes.
Refinement. Refinement of *F*^2^ against ALL reflections. The weighted *R*-factor wR and goodness of fit *S* are based on *F*^2^, conventional *R*-factors *R* are based on *F*, with *F* set to zero for negative *F*^2^. The threshold expression of *F*^2^ > σ(*F*^2^) is used only for calculating *R*-factors(gt) etc. and is not relevant to the choice of reflections for refinement. *R*-factors based on *F*^2^ are statistically about twice as large as those based on *F*, and *R*- factors based on ALL data will be even larger.

### Fractional atomic coordinates and isotropic or equivalent isotropic displacement parameters (Å^2^)

**Table:**

	*x*	*y*	*z*	*U*_iso_*/*U*_eq_	
Br1	0.58913 (3)	0.68699 (4)	1.13810 (7)	0.0709 (3)	
C1	−0.0081 (3)	1.0150 (5)	0.1918 (5)	0.0559 (11)	
H1	−0.0372	0.9491	0.1526	0.067*	
C2	0.0624 (3)	1.0028 (4)	0.3238 (5)	0.0450 (9)	
C3	0.1048 (3)	1.0973 (3)	0.3971 (5)	0.0437 (9)	
H3	0.0915	1.1693	0.3572	0.052*	
C4	0.1657 (3)	1.0851 (3)	0.5270 (4)	0.0409 (9)	
C5	0.1854 (3)	0.9748 (3)	0.5878 (5)	0.0411 (9)	
C6	0.1453 (3)	0.8818 (4)	0.5123 (5)	0.0483 (10)	
H6	0.1598	0.8093	0.5493	0.058*	
C7	0.0832 (3)	0.8959 (4)	0.3805 (5)	0.0492 (10)	
H7	0.0556	0.8328	0.3305	0.059*	
C8	0.1941 (3)	1.2830 (4)	0.5499 (6)	0.0532 (11)	
H8A	0.2168	1.2873	0.4502	0.080*	
H8B	0.2278	1.3363	0.6180	0.080*	
H8C	0.1282	1.3007	0.5412	0.080*	
C9	0.2625 (3)	0.8629 (3)	0.7868 (5)	0.0561 (12)	
H9A	0.2991	0.8176	0.7216	0.067*	
H9B	0.2040	0.8234	0.7983	0.067*	
C10	0.3167 (3)	0.8807 (3)	0.9420 (5)	0.0446 (10)	
C11	0.4120 (3)	0.8598 (3)	0.9632 (5)	0.0482 (10)	
H11	0.4430	0.8359	0.8802	0.058*	
C12	0.4628 (3)	0.8737 (3)	1.1059 (5)	0.0486 (10)	
C13	0.4153 (4)	0.9079 (3)	1.2281 (5)	0.0576 (12)	
H13	0.4481	0.9167	1.3247	0.069*	
C14	0.3197 (3)	0.9293 (4)	1.2093 (6)	0.0581 (12)	
H14	0.2886	0.9527	1.2925	0.070*	
C15	0.2709 (3)	0.9158 (3)	1.0667 (6)	0.0530 (11)	
H15	0.2065	0.9303	1.0537	0.064*	
C16	0.5658 (3)	0.8494 (4)	1.1234 (7)	0.0683 (14)	
H16A	0.5948	0.8865	1.2153	0.082*	
H16B	0.5942	0.8798	1.0356	0.082*	
O1	−0.0319 (2)	1.1038 (3)	0.1288 (4)	0.0668 (9)	
O2	0.2083 (2)	1.1717 (2)	0.6096 (3)	0.0501 (7)	
O3	0.2435 (2)	0.9726 (2)	0.7192 (3)	0.0516 (7)	

### Atomic displacement parameters (Å^2^)

**Table:**

	*U*^11^	*U*^22^	*U*^33^	*U*^12^	*U*^13^	*U*^23^
Br1	0.0595 (3)	0.0524 (3)	0.0992 (5)	0.0119 (2)	0.0002 (3)	0.0131 (3)
C1	0.056 (3)	0.067 (3)	0.045 (3)	−0.005 (2)	0.003 (2)	−0.007 (2)
C2	0.040 (2)	0.056 (2)	0.040 (2)	−0.0003 (18)	0.0050 (18)	−0.003 (2)
C3	0.047 (2)	0.041 (2)	0.044 (2)	0.0044 (17)	0.0074 (19)	0.0016 (19)
C4	0.041 (2)	0.039 (2)	0.043 (2)	0.0013 (16)	0.0025 (18)	−0.0020 (18)
C5	0.041 (2)	0.040 (2)	0.042 (2)	0.0006 (16)	0.0021 (18)	−0.0009 (18)
C6	0.053 (2)	0.038 (2)	0.053 (3)	0.0029 (18)	0.004 (2)	0.0008 (19)
C7	0.049 (2)	0.048 (2)	0.050 (2)	−0.0055 (19)	0.002 (2)	−0.010 (2)
C8	0.063 (3)	0.039 (2)	0.055 (3)	0.0037 (19)	−0.007 (2)	0.000 (2)
C9	0.071 (3)	0.033 (2)	0.061 (3)	0.005 (2)	−0.011 (2)	0.007 (2)
C10	0.052 (2)	0.0263 (18)	0.054 (3)	0.0026 (16)	0.001 (2)	0.0082 (18)
C11	0.051 (2)	0.036 (2)	0.058 (3)	0.0052 (18)	0.008 (2)	0.006 (2)
C12	0.051 (2)	0.0295 (19)	0.064 (3)	0.0006 (17)	−0.002 (2)	0.009 (2)
C13	0.083 (3)	0.034 (2)	0.052 (3)	−0.001 (2)	−0.011 (2)	0.003 (2)
C14	0.072 (3)	0.039 (2)	0.064 (3)	0.001 (2)	0.016 (3)	−0.003 (2)
C15	0.050 (2)	0.033 (2)	0.075 (3)	0.0017 (18)	0.005 (2)	0.001 (2)
C16	0.058 (3)	0.046 (3)	0.098 (4)	−0.002 (2)	−0.009 (3)	0.013 (3)
O1	0.063 (2)	0.078 (2)	0.055 (2)	0.0041 (18)	−0.0122 (16)	0.0038 (19)
O2	0.0631 (18)	0.0365 (15)	0.0474 (17)	−0.0004 (13)	−0.0112 (14)	0.0001 (12)
O3	0.0621 (18)	0.0359 (14)	0.0536 (18)	0.0014 (13)	−0.0108 (15)	0.0060 (13)

### Geometric parameters (Å, °)

**Table:**

Br1—C16	1.946 (5)	C8—H8C	0.9600
C1—O1	1.216 (6)	C9—O3	1.437 (5)
C1—C2	1.460 (6)	C9—C10	1.508 (6)
C1—H1	0.9300	C9—H9A	0.9700
C2—C7	1.376 (6)	C9—H9B	0.9700
C2—C3	1.393 (6)	C10—C11	1.376 (6)
C3—C4	1.367 (6)	C10—C15	1.388 (6)
C3—H3	0.9300	C11—C12	1.388 (6)
C4—O2	1.358 (5)	C11—H11	0.9300
C4—C5	1.422 (5)	C12—C13	1.380 (6)
C5—O3	1.348 (5)	C12—C16	1.492 (6)
C5—C6	1.375 (6)	C13—C14	1.381 (7)
C6—C7	1.392 (6)	C13—H13	0.9300
C6—H6	0.9300	C14—C15	1.375 (7)
C7—H7	0.9300	C14—H14	0.9300
C8—O2	1.420 (5)	C15—H15	0.9300
C8—H8A	0.9600	C16—H16A	0.9700
C8—H8B	0.9600	C16—H16B	0.9700
			
O1—C1—C2	125.6 (4)	O3—C9—H9B	110.2
O1—C1—H1	117.2	C10—C9—H9B	110.2
C2—C1—H1	117.2	H9A—C9—H9B	108.5
C7—C2—C3	120.0 (4)	C11—C10—C15	118.9 (4)
C7—C2—C1	118.7 (4)	C11—C10—C9	120.6 (4)
C3—C2—C1	121.2 (4)	C15—C10—C9	120.4 (4)
C4—C3—C2	120.6 (4)	C10—C11—C12	121.4 (4)
C4—C3—H3	119.7	C10—C11—H11	119.3
C2—C3—H3	119.7	C12—C11—H11	119.3
O2—C4—C3	125.2 (4)	C13—C12—C11	118.4 (4)
O2—C4—C5	115.3 (3)	C13—C12—C16	122.2 (5)
C3—C4—C5	119.5 (4)	C11—C12—C16	119.4 (4)
O3—C5—C6	125.8 (4)	C12—C13—C14	121.1 (4)
O3—C5—C4	114.7 (3)	C12—C13—H13	119.4
C6—C5—C4	119.5 (4)	C14—C13—H13	119.4
C5—C6—C7	120.1 (4)	C15—C14—C13	119.6 (4)
C5—C6—H6	119.9	C15—C14—H14	120.2
C7—C6—H6	119.9	C13—C14—H14	120.2
C2—C7—C6	120.3 (4)	C14—C15—C10	120.6 (4)
C2—C7—H7	119.9	C14—C15—H15	119.7
C6—C7—H7	119.9	C10—C15—H15	119.7
O2—C8—H8A	109.5	C12—C16—Br1	110.9 (3)
O2—C8—H8B	109.5	C12—C16—H16A	109.5
H8A—C8—H8B	109.5	Br1—C16—H16A	109.5
O2—C8—H8C	109.5	C12—C16—H16B	109.5
H8A—C8—H8C	109.5	Br1—C16—H16B	109.5
H8B—C8—H8C	109.5	H16A—C16—H16B	108.1
O3—C9—C10	107.6 (3)	C4—O2—C8	117.4 (3)
O3—C9—H9A	110.2	C5—O3—C9	116.2 (3)
C10—C9—H9A	110.2		
			
O1—C1—C2—C7	−178.7 (4)	C15—C10—C11—C12	−0.4 (6)
O1—C1—C2—C3	4.6 (7)	C9—C10—C11—C12	−178.8 (4)
C7—C2—C3—C4	−1.3 (6)	C10—C11—C12—C13	0.8 (6)
C1—C2—C3—C4	175.4 (4)	C10—C11—C12—C16	179.7 (4)
C2—C3—C4—O2	−177.8 (4)	C11—C12—C13—C14	−0.8 (6)
C2—C3—C4—C5	−0.3 (6)	C16—C12—C13—C14	−179.7 (4)
O2—C4—C5—O3	0.2 (5)	C12—C13—C14—C15	0.3 (6)
C3—C4—C5—O3	−177.6 (3)	C13—C14—C15—C10	0.1 (6)
O2—C4—C5—C6	179.9 (4)	C11—C10—C15—C14	−0.1 (6)
C3—C4—C5—C6	2.1 (6)	C9—C10—C15—C14	178.3 (4)
O3—C5—C6—C7	177.3 (4)	C13—C12—C16—Br1	100.3 (5)
C4—C5—C6—C7	−2.5 (6)	C11—C12—C16—Br1	−78.6 (5)
C3—C2—C7—C6	1.0 (6)	C3—C4—O2—C8	−5.1 (6)
C1—C2—C7—C6	−175.8 (4)	C5—C4—O2—C8	177.3 (4)
C5—C6—C7—C2	0.9 (6)	C6—C5—O3—C9	−1.7 (6)
O3—C9—C10—C11	−104.6 (4)	C4—C5—O3—C9	178.0 (3)
O3—C9—C10—C15	77.0 (5)	C10—C9—O3—C5	−172.1 (3)
